# Human usage in the native range may determine future genetic structure of an invasion: insights from *Acacia pycnantha*

**DOI:** 10.1186/1472-6785-13-37

**Published:** 2013-10-01

**Authors:** Johannes J Le Roux, David M Richardson, John RU Wilson, Joice Ndlovu

**Affiliations:** 1 Centre for Invasion Biology. Department of Botany and Zoology, Stellenbosch University, Private Bag X1, Matieland 7602, South Africa; 2 South African National Biodiversity Institute, Kirstenbosch National Botanical Gardens, Claremont 7735, South Africa

**Keywords:** *Acacia pycnantha*, Admixture, Biological invasions, Genetic structure, Native range, Restoration, Wattle

## Abstract

**Background:**

The influence of introduction history and post-introduction dynamics on genetic diversity and structure has been a major research focus in invasion biology. However, genetic diversity and structure in the invasive range can also be affected by human-mediated processes in the native range prior to species introductions, an aspect often neglected in invasion biology. Here we aim to trace the native provenance of the invasive tree *Acacia pycnantha* by comparing the genetic diversity and structure between populations in the native Australian range and the invasive range in South Africa. This approach also allowed us to explore how human actions altered genetic structure before and after the introduction of *A. pycnantha* into South Africa. We hypothesized that extensive movement and replanting in *A. pycnantha*’s Australian range prior to its introduction to South Africa might result in highly admixed genotypes in the introduced range, comparable genetic diversity in both ranges, and therefore preclude an accurate determination of native provenance(s) of invasive populations.

**Results:**

In the native range Bayesian assignment tests identified three genetic clusters with substantial admixture and could not clearly differentiate previously identified genetic entities, corroborating admixture as a result of replantings within Australia. Assignment tests that included invasive populations from South Africa indicated similar levels of admixture compared to Australian populations and a lack of genetic structure. Invasive populations of *A. pycnantha* in South Africa are as genetically diverse as native populations, and could not be assigned to particular native range regions.

**Conclusions:**

Our results indicate that the genetic structure of *A. pycnantha* in Australia has been greatly altered through various planting initiatives. Specifically, there is little geographic structure and high levels of admixture. While numerous introduction history scenarios may explain the levels of admixture observed in South Africa, planting records of *A. pycnantha* in Australia suggest that populations were probably already admixed before propagules were introduced to South Africa. These findings have important implications for the management of invasive *A. pycnantha* populations in South Africa, especially for classical biological control, and more broadly, for studies that aim to understand the evolutionary dynamics of the invasion process.

## Background

The effect of invasion history on genetic diversity has been documented for numerous species e.g. [1-3]. Many successful invasions are characterized by high genetic diversity – the result of multiple introductions or high propagule pressure from a single source [4], but other invasions are founded by genetically bottlenecked populations which harbour only a small proportion of the total genetic diversity found in their native ranges [5]. Intuitively, high genetic diversity is likely to be beneficial to any species introduced into a new environment. Multiple introductions from distinct native source populations can cause an immediate breakdown of natural gene flow barriers, often leading to admixture (e.g. [6]), increased population genetic diversity [6] hybridization [7] and even genetic novelty [8] in the invaded range. High propagule pressure may also simply enhance the likelihood of introducing suitable genotypes to the new environment [9]. However, a dramatic reduction in genetic diversity, and therefore evolutionary potential, need not limit an invasion, as release from natural enemies [10] and competitors [11], broad environmental tolerance [12], and pre-adaptations [13] may contribute to the success of an invasion.

Disentangling the deterministic and stochastic processes that underlie the genetic diversity and structure of successful invaders is problematic [14], but has important implications for the effectiveness of management interventions, particularly biological control (e.g. [15]). While introduction and post-introduction dynamics have important roles, the genetic structure in the native range population can also influence the way in which introduction histories impact on genetic diversity found in the invasive range. For example, for highly structured populations multiple introduction events from a single population may lead to lower overall diversity than a single introduction sourced from numerous structured native range populations [16].

Tree species introduced for forestry represent a particularly interesting case to explore genetic diversity and structure. These species are typically sampled over large parts of the native range and in large numbers prior to introduction in order to maximize genetic diversity, environmental sampling, and thus evolutionary/breeding potential [16]. Extensive breeding programmes in both the native and introduced range might also ‘pre-adapt’ introduced entities to local environmental conditions [16]. Consequently, the selection, introduction, and establishment of forestry species is often associated with traits linked to successful invasions: high propagule pressure, short generation times, high growth rates [9,17], as well as high adaptability as mediated through high genetic diversity [4,6,8,18,19].

Given the complex introduction histories often associated with forestry species, elucidating the processes that shape genetic diversity in the invasive ranges requires accurate and detailed introduction records [16]. Such records are available for the introduction of *Acacia pycnantha* (Benth.) to South Africa [20]. *Acacia pycnantha*, commonly known as the golden wattle, is native to south eastern Australia and was introduced to South Africa on two separate occasions in 1865 and 1893 as a potential source of tanbark and for dune reclamation purposes [20]. Experimental plantings of *A. pycnantha* showed the species to be a promising candidate for tanbark production. While the exact size of both introduction events is unknown, the redistribution of ca. 22–29 million seeds sourced within South Africa throughout the coastal regions of the country is documented [20,21]. Like many other Australian acacias, *A. pycnantha* is now invasive in parts of South Africa [22].

In its native range, *A. pycnantha* is structured into two distinct ecotypes (the dryland and wetland forms) which show a propensity for hybridization [23]. South African populations are genetically less diverse than Australian populations and most closely resemble the wetland form from southern Australia [23]. However, revegetation and roadside plantings from cultivated plants have led to established populations of *A. pycnantha* in southern Australia and this may obscure phylogeographic signatures in the native range. Given the known impacts of cultivation on the genetic makeup of invasive species, including Australian acacias [8], our overall aim was to use population genetics approaches to better understand the native source(s), genetic diversity, structure, and dynamics of invasive *A. pycnantha* populations in South Africa. Specifically, we asked: 1) What is the genetic structure in the native range of *A. pycnantha?* 2) How much of the genetic diversity in *A. pycnantha*’s native range has been introduced to South Africa? 3) Can source populations of *A. pycnantha* invading South Africa be identified? 4) Does admixture of geographically isolated genotypes from Australia occur in South Africa?

## Results

### Genetic diversity

There was evidence of null alleles at one locus (Plop 18) in eight of the populations and so this locus was excluded in further analyses. Tests for Hardy Weinberg equilibrium (HWE) carried out on the remaining seven microsatellites showed that 153 out of 168 locus-by-site comparisons met expectations under HWE. Fifteen locus-by-site comparisons showed an excess of homozygotes. Of these, only three populations out of the 24 sampled populations showed more than one locus deviating from HWE. Three loci (As 2.17, Am435 and Plop 4) were out of HWE for at least three sampled populations. However, in these cases homozygote excess was not attributed to the presence of null alleles in the dataset.

Overall, the number of alleles (*N*_*A*_), unbiased genetic diversity (*H*_*S*_), and allelic richness (*R*_*S*_) were not significantly different in the native range (Australia) than in the invasive range (South Africa). Furthermore, populations in the native range were slightly more structured than populations in the invasive range (Table 1). Overall, *A. pycnantha* showed very little to no inbreeding in both its native and introduced ranges (Tables 1 and 2).

**Table 1:** **Comparison of genetic diversity indices between native (Australian) and invasive (South African) populations of *Acacia pycnantha***

**Region**	***R***_**S**_	***H***_**S**_	***H***_**O**_	***F***_**IS**_	***F***_**ST**_
Native (Australia)	2.43	0.636	0.685	-0.078	0.084
Invasive (South Africa)	2.35	0.608	0.649	-0.067	0.051

*Abbreviations*: *R*_S_ allelic richness, *H*_S_ unbiased gene diversity, *H*_**O**_ observed heterozygosity, *F*_IS_ inbreeding coefficient, *F*_ST_ among-population differentiation. None of the comparisons were significantly different between South African and Australian populations.

**Table 2:** **Genetic diversity indices at seven microsatellite loci and 24 populations (17 native and 7 invasive) of *Acacia pycnantha***

**Sample ID**	**Latitude**	**Longitude**	**N**	***N***_**a**_	***H***_**O**_	***H***_**E**_	***F***_**IS**_	***N***_**P**_
**Australia (native)**								
Mt Compass (MTC)	-35.40558	138.599882	19	6.43	0.73	0.67	-0.061	0
Melrose (MEL)	-32.78187	138.1973	28	6.43	0.63	0.69	0.105	3
Kilmore (KIL)	-37.22176	145.021	26	5.57	0.58	0.58	0.018	0
Natimuk (NAT)	-36.00409	143.76041	26	6.29	0.68	0.65	-0.024	3
Frances (FRA)	-36.77054	141.18135	25	5.42	0.70	0.65	-0.066	0
Border (NSW/VIC)	-35.83107	147.22716	29	7.29	0.78	0.69	-0.125	1
Charlton & Boorte (CB)	-35.99273	143.76538	28	6.14	0.80	0.68	-0.219	4
Mt Jerrabomberra (MTJ)	-35.36866	149.20332	21	4.86	0.63	0.52	-0.188 0	
Lockhart (LOC)	-35.36866	146.64549	21	4.86	0.72	0.64	-0.085	1
Gundagai (GUN)	-35.21065	147.76425	22	4.71	0.60	0.52	-0.011	0
Reef Hills (RHSP)	-36.59888	145.95586	22	5.57	0.61	0.61	0.026	0
Kangaroo Island (KIS)	-35.75669	137.89486	5	4.29	0.74	0.75	0.01	0
Newlands C.P. (NLHCP)	-35.61298	138.47950	5	3.33	0.74	0.67	-0.22	0
Nelson (NEL)	-38.05003	141.01510	8	3.71	0.63	0.54	-0.248	0
Castlemaine (CAS)	-37.10758	144.09283	5	3.43	0.61	0.51	-0.05	0
Murray Bridge (MB)	-35.32020	139.51302	5	3.57	0.67	0.51	-0.054	0
Hall’s Gap (HG)	-37.11027	142.57697	6	3.71	0.70	0.65	-0.091	0
**Average**				**3.55**	**0.67**	**0.58**	**-0.133**	
**South Africa (invasive)**								
Caledon (CAL)	-34.21954	19.42565	27	3.86	0.59	0.52	-0.134	0
Grahamstown (GRT)	-33.46032	26.15991	25	5.86	0.58	0.56	-0.013	2
Tokai (TOK)	-33.84179	18.66602	28	4.86	0.55	0.59	0.072	0
Humansdorp (HUM)	-34.03989	-24.78687	18	6.00	0.70	0.62	-0.086	1
Wolsely (WOL)	-33.34012	19.16109	26	6.43	0.72	0.65	-0.095	1
Stellenrust (STE)	-34.06024	18.41480	27	6.00	0.69	0.61	-0.117	2
Piketberg (PIK)	-32.80084	18.71501	21	4.86	0.72	0.64	-0.09	0
**Average**				**5.41**	**0.65**	**0.60**	**-0.066**	

*Abbreviations*: *N* number of individuals per population, *N*_a_ number of alleles, *H*_O_ observed heterozygosity, *H*_E_ expected heterozygosity, *F*_IS_ inbreeding coefficient and *N*_P_ number of private alleles.

For levels of intra-population genetic diversities, the mean number of alleles was slightly higher in the introduced populations than in the native range (Table 2). Similarly, expected heterozygosity (*H*_E_) was slightly higher in the invaded range than in the native range (Table 2). Fewer private alleles (*N*_P_) were found in the invaded ranges in South Africa than in the native range (Table 2).

### Genetic structure

**Figure 1:**
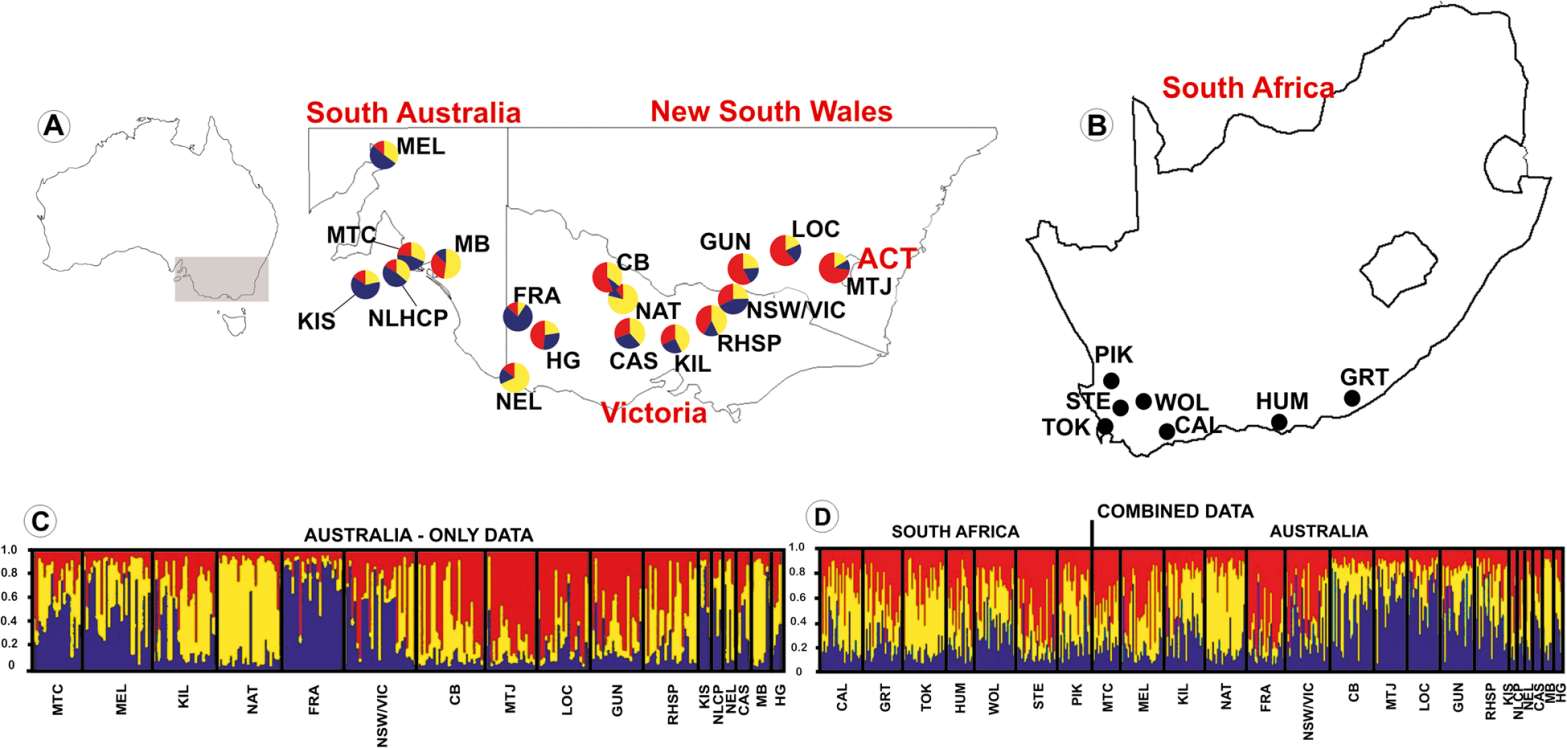
**Population genetic structure based on Bayesian assignment tests performed in STRUCTURE. (A)** Localities in south eastern (SE) Australia where native *Acacia pycnantha* populations were sampled. Pie charts indicate overall genotype assignment for each population to particular genetic clusters identified based on native range data only. **(B)** Localities in South Africa where invasive *Acacia pycnantha* populations were sampled. **(C)** Results of the STRUCTURE analysis showing population genetic structure of *A. pycnantha* populations in its native range (based on native range data only) and **(D)** combined native (SE Australia) and invasive (South Africa) ranges (combined data). The vertical axes of all STRUCTURE bar plots illustrate the proportional assignment of individual genomes to the inferred genetic groups.

Bayesian assignment analyses in STRUCTURE revealed three different genetic clusters as the optimal number among native range populations while four genetic clusters were identified for the combined native and invasive range datasets (Figure 1 and Additional file 1: Figure S1). For the native range data the three identified genetic clusters only roughly corresponded to three distinct regions from east to west across *A. pycnantha’s* natural range, including South Australia and Flinders Range (cluster 3, indicated in blue in Figure 1A), the drier parts of Victoria (cluster 2, indicated in yellow in Figure 1A), and New South Wales and some wetter parts of Victoria (cluster 1, indicated in red in Figure 1A). However, the majority of native range populations could not be confidently assigned to any single cluster (q > 0.8), indicative of extensive admixture. Our results also indicate that South African invasive populations probably originated from seeds collected from the entire native range distribution in Australia (Figure 1D). Although the invasive *A. pycnantha* populations may therefore also represent genetic material from the Flinders Range (dryland form of *A. pycnantha,* population MEL) it is highly unlikely that this region is a putative source region because of the marked differences observed in leaf morphologies that correspond to distinct ecotypes not found in South Africa [23]. This is supported by chloroplast DNA sequence data obtained from the Flinders Range population that showed no similarity to South African invasive populations [23].

**Figure 2:**
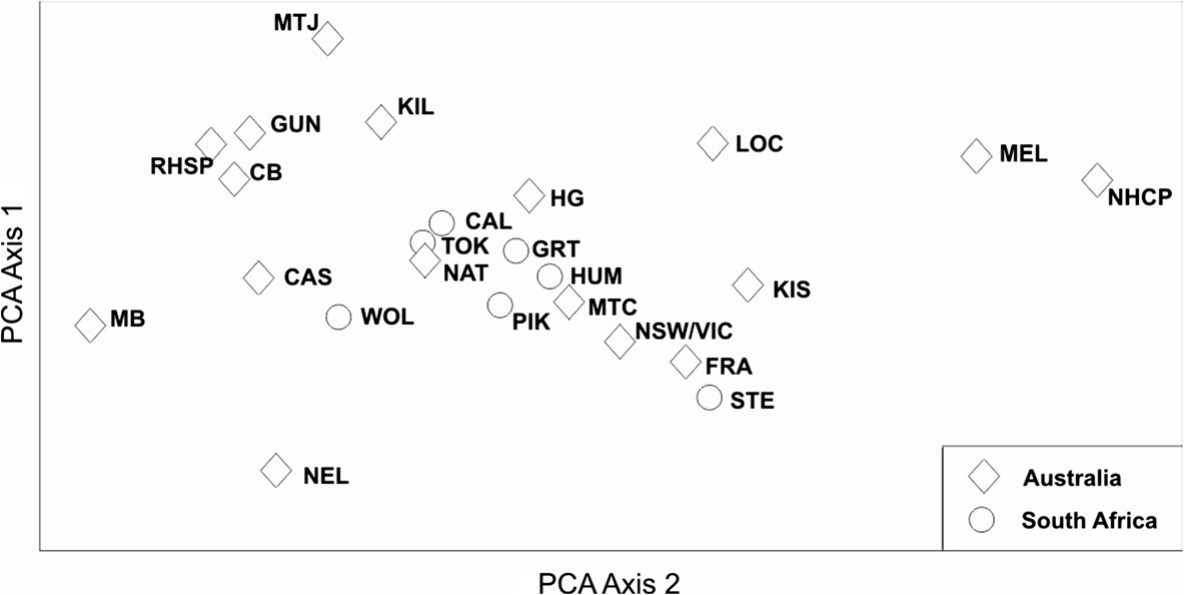
**Plot of the first two axes of a PCoA showing genetic differentiation based on pairwise Φ**_**PT **_**values for native (Australian) and invasive (South African) populations of *Acacia pycnantha*.**

Hierarchical AMOVA of all samples showed almost no differentiation between the native and invasion ranges (1%) but considerable differentiation among populations (13%) whereas the majority of genetic variation resided within populations (86%) (Table 3). The PCoA indicated a close relationship between invasive *A. pycnantha* from South Africa and native populations from southern Australia, particularly Mt Compass (population MTC) and some Victorian populations (populations HG and NAT) (Figure 2).

**Table 3:** **Hierarchical analysis of molecular variance (AMOVA) of genetic diversity among native and invasive regions (Australia and South Africa), among populations, and within populations of *Acacia pycnantha***

**Source of variation**	**df**	**Sum of squares**	**Variance**	**Percent variation (%)**
Among native and invasive regions	1	33.28	0.049	1
Among populations	22	418.6	0.726	13
Within populations	449	2240.3	4.99	86

## Discussion and conclusions

We found very low population genetic structure and high levels of gene flow throughout *A. pycnantha*’s native distribution in south eastern Australia (Figure 1A and C). While this observation supports a previous phylogeographic analysis indicating that admixture frequently occurs in Australian populations, it is also in contrast to the genetic structure previously identified between different ecotypes (dry and wetland forms) of *A. pycnantha.* These two forms have been estimated to have diverged during the Pleistocene, around 100 KYA [23]. Here we included individuals collected from the same populations as those reported previously on by Ndlovu *et al*. [23].

Our results make sense as *A. pycnantha* is known to have been widely moved and planted throughout Australia [24]. Trial plantations have been established at numerous sites in Australia due to the species’ hardiness, drought tolerance and good performance under a range of soil conditions [24]. It has also been widely used for revegetation and soil stabilisation as it shows high natural colonising ability and fast growth under field conditions (http://www.treesforlife.org.au). Like many other wattles, *A. pycnantha* was heavily harvested in the wild as early as the 1840s. The unsustainable harvesting of wattle bark from natural populations prompted authorities in Victoria to appoint a Wattle Bark Board in 1878 [25]. This Board recommended the establishment of wattle plantations as a sustainable source of tannin bark; these were established in New South Wales, South Australia, Tasmania and Victoria between 1880 and 1900 [26]. For example, the Woods and Forests Department Annual Report (South Australia) for the years 1883–84 [27] mentions that around 40 acres of *A. pycnantha* plantation were established at Bundaleer, South Australia; during this period testing and dissemination of both Australian native species and alien species was well underway within Australia (D Bush, Australian Tree Seed Centre, CSIRO Plant Industry, personal communication). These practices may have resulted in substantial mixing of genetic material in *A. pycnantha*’s native range, providing many opportunities for interbreeding to occur between previously allopatric populations [24]. On the other hand, the extensive movement of *A. pycnantha* within South Africa following introduction may also explain high levels of admixture observed in the invasive range, but it seems more likely that seeds introduced to South Africa in 1893 came from already admixed sources [20].

It is also conceivable that the lack of genetic structure and high levels of admixture observed for *A. pycnantha* in Australia simply reflects a species with high levels of gene flow. We consider this unlikely, as numerous authors have previously noted that native *A. pycnantha* populations appear to be structured into two distinct ecotypes [24,28] and that deep genetic divergence exists between the two [23]. Also, most acacias investigated to date show moderate to high levels of population genetic structure over various spatial scales (e.g. [8,29,30]) - including species that co-occur with *A. pycnantha* in its native range, like *A. mearnsii*[31] and *A. melanoxylon*[32].

The role of genetic admixture in successful establishment and invasion has been documented for numerous species [3,33,34]. This study is, however, as far as we know, the first to show that anthropogenic actions (plantings for revegetation and the establishment of plantations for bark in this case) may lead to admixture in the native range prior to a species’ introduction into its new range. Of course high levels admixture in South Africa may have also resulted from multiple introductions followed by the extensive redistribution of seed stocks within the country [20,21]. However, forestry records indicate that the movement and planting of *A. pycnantha* within Australia predates its introduction to South Africa [26,27], making a strong case for native range admixture prior to introduction to South Africa. Admixture may enhance the invasion success of a species through pre-adaptation to a wider range of bioclimatic conditions prior to introduction to new regions [35]. Furthermore, because admixture in the native range may increase overall genetic diversity, the likelihood of introducing highly diverse genotypes is enhanced, which also improves the chances of invasion success. Other benefits of admixture to invasion success include masking of deleterious alleles and the creation of novel genotypes [36].

Since the Australian landscape is characterised by many revegetated forests, it is difficult to identify putative sources of invasive *A. pycnantha* because admixed propagules of *A. pycnantha* have most likely been introduced to South Africa. However, separate introductions of the biological control agent, the gall-forming wasp *Trichilogaster signiventris,* from the dryland (Natimuk, Victoria) and the wetland regions of *A. pycnantha* (Mt Compass) had differential success in South Africa. Such differences in biocontrol efficacy can be indicative of native provenance. It would be useful to test this hypothesis, in line with other work on the influence of phylogeographic structure on the efficacy of biological control (e.g. [15]), as part of the ongoing efforts to improve the contribution of classical biological control to the integrated control of Australian acacias in South Africa [37].

The *Acacia pycnantha* case demonstrates how human influences on native range population genetic structure prior to a species being even considered for introduction, might ultimately influence evolutionary potential in the introduced range. This is an important but hitherto overlooked dimension of species introduction histories that deserves careful consideration in future studies aimed at better understanding the evolutionary consequences of invasive species.

## Methods

### Sample collection

Phyllode material of *A. pycnantha* was collected at seventeen sites throughout the native range (south eastern Australia) and at seven sites from the invaded ranges in South Africa (Table 1). Material was also collected from Mt Compass in South Australia from where the biocontrol agent *Trichilogaster signiventris* which has successfully established in South Africa was previously collected [38]. For each site, material was collected from between five and 30 trees and preserved in silica gel until DNA extraction. Collection sites were geo-referenced using a handheld GPS.

### DNA extraction and PCR conditions

DNA was extracted from phyllode material using a modified [23] CTAB extraction protocol [39]. DNA concentrations were measured using a spectrophotometric nanodrop (Infinite 200 PRO NanoQuant, Tecan Group Ltd, Switzerland) and good quality DNA diluted to 20 ng/μL and stored at −80°C until further use. Eight nuclear microsatellite markers that were previously developed for *Acacia mangium*[40], *A. saligna*[41] and *Paraserianthes lophantha*[42] were cross-amplified in all individuals (see Additional file 2: Table S1). PCR was conducted in two separate multiplexes which were performed using the Qiagen multiplex PCR kit following the manufacturer’s instructions: 5 μL of 2× Qiagen mix, 2 μL of primer mix (containing 2 μM of each primer), 2 μL of RNase free water and 1 μL of DNA template to make up a final volume of 10 μL. The following thermocycle was used: an initial denaturation step of 95°C for 15 min followed by 35 cycles of 94°C for 30 s, an annealing temperature of 57°C for 90s and 72°C for 60 s. A final elongation step of 60°C for 30 min was performed. Separation of PCR fragments was done on an ABI Prism 3100 Genetic Analyzer (Applied Biosystems, Foster City, USA) using GENESCAN TM- 500 (−250) as an internal size standard. Allele scoring was done using GENEMARKER version 1.95 (SoftGenetics LLC, Pennsylvania, USA).

### Data analysis

#### Genetic diversity

Microchecker version 2.2.3 [43] was used to check for null alleles, large allele dropouts and allelic stutter. In addition, FreeNA [44] was also used to examine the presence of null allele frequencies for each locus and population following the expectation maximisation algorithm. All microsatellite loci were tested for departures from the Hardy-Weinberg equilibrium and linkage disequilibrium using the Adegenet package [45] in the *R* statistical environment [46]. ARLEQUIN version 3.5.1.2 [47] was used to calculate the number of alleles (*N*_A_), allelic richness (*R*_*S*_), observed and expected heterozygosities (*H*_O_ and *H*_E_), fixation indices (*F*_ST_) and inbreeding coefficients (*F*_IS_) for invasive and native ranges and within populations. The mean number of private alleles (*N*_*P*_*)* per population was computed in GenAlex v. 6 [48]. Statistical comparisons to evaluate differences in the genetic diversity indices were calculated using permutation procedures as implemented in using FSTAT version 2.9.3.2 [49].

### Genetic structure

Bayesian clustering methods implemented in the programme STRUCTURE version 2.3.4 [50] were used to detect the number of genetic clusters (*K*) present in both the native range and the combined native and invasive range datasets, and to assign individuals probabilistically to these clusters (for *K* = 1–10). For both analyses, the admixture model with correlated allele frequencies was chosen and 10 replicates of each value of *K* were run. Each run consisted of a burnin of 10000 MCMC steps, followed by 1000000 iterations. The method of Evanno *et al.*[51] was used to determine the optimal number of genetic clusters.

To assess the distribution of genotypes in the native and invasive ranges a covariance standardised Principal Co-ordinate Analysis (PCoA) implemented in GenAlex version 6 [48] was used. An analysis of molecular variance (AMOVA) [47] was also performed to partition genetic variation between regions and among regions.

## Competing interests

The authors declare that they have no competing interests.

## Authors’ contributions

All authors developed the project and participated in field collections. JLR and JN analysed the data and led the writing of the manuscript. All authors were involved in data interpretation and writing and read, edited, and approved the final manuscript.

## Supplementary Material

Additional file 1: Figure S1 Plots of the rate of change (delta K) based on STRUCTURE results. Click here for file

Additional file 2: Table S1 Polymorphic microsatellites and multiplexes used in this study. Click here for file
