# Supplementary material for: Human usage in the native range may determine future genetic structure of an invasion: insights from Acacia pycnantha
Source: BMC Ecol. 2013 Oct 1;13:37. doi: 10.1186/1472-6785-13-37 (PMC3840604; doi:10.1186/1472-6785-13-37)
Supplement: Additional file 1: Figure S1 — Plots of the rate of change (delta K) based on STRUCTURE results. [file 1472-6785-13-37-S1.docx]

Online supplementary information:


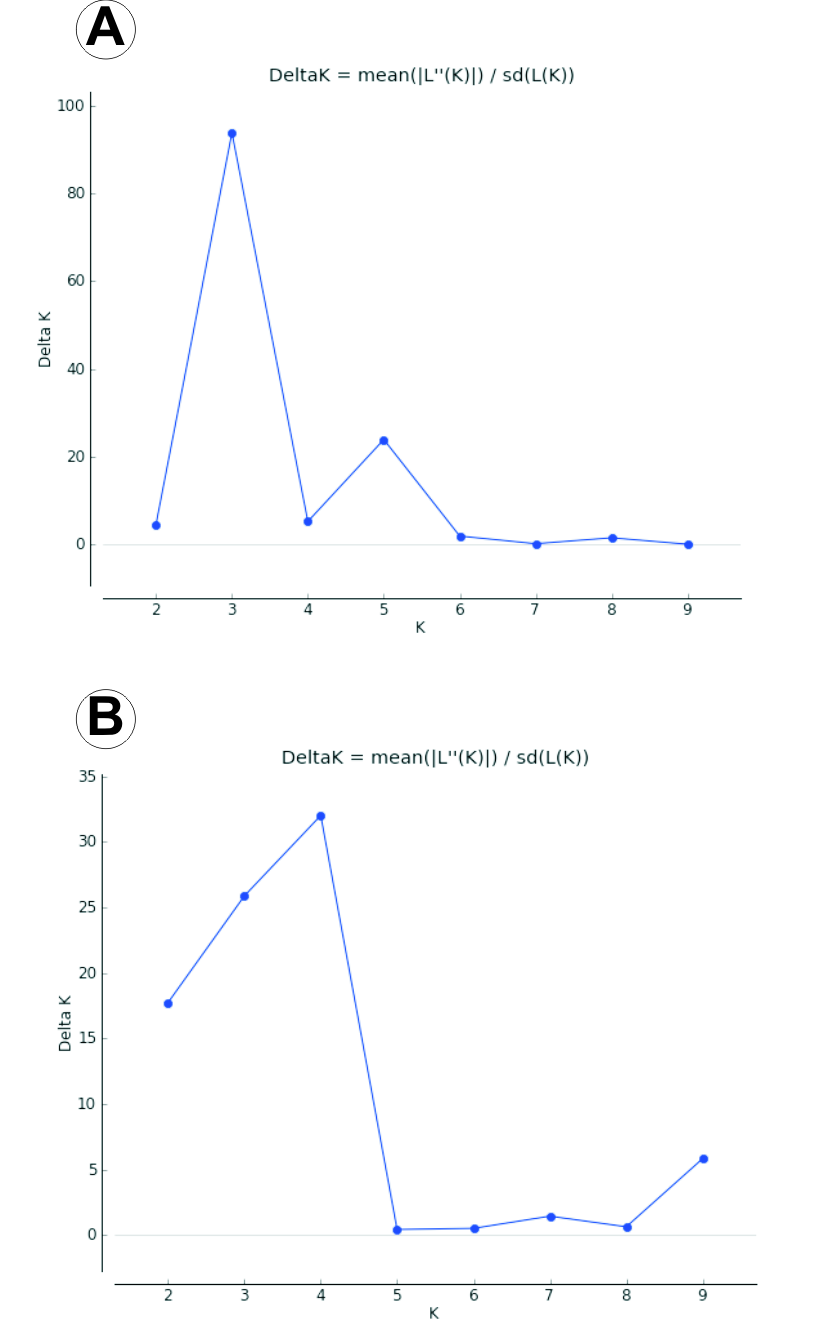


Figure S1: Plots of the rate of change (delta K) based on 10 replicates per *K* generated by the STRUCTURE program [50] according to the method of Evanno et al. [51] for A) Australian data only and B) Combined Australian and South African data.
