# Supplementary material for: Human usage in the native range may determine future genetic structure of an invasion: insights from Acacia pycnantha
Source: BMC Ecol. 2013 Oct 1;13:37. doi: 10.1186/1472-6785-13-37 (PMC3840604; doi:10.1186/1472-6785-13-37)
Supplement: Additional file 2: Table S1 — Polymorphic microsatellites and multiplexes used in this study. [file 1472-6785-13-37-S2.doc]

Additional file 2: Table S1 Polymorphic microsatellites and multiplexes used in this study

**Primer name Microsatellite sequence Fluorescent label Size Multiplex**

Am352 **F:** CCTCATGTCCTTGAATGTCAC 6 – FAM 127-129 1

**R:** GACTAACCCACAAGGAAGAGTTAC

Am429 **F:** CCTTCTTCTCTCATCTACCAAACC PET 170-180 1

**R:** CCCACATCATCACTCACAACT

Am435 **F:** ACCCTTTATTTCTCACACGGA 6 – FAM 139-152 2

**R:** ACAGAAGAAGATGCAAAGAAGG

Am436 **F:** ATGGATCTTGTCCTTATCTTGA VIC 240-246 1

**R:** GGGCCAATTTGAGTTTGGAA

Am502 **F:** CAAATGGCCAAGTTACGACTG VIC 122-128 1

**R:** TTCTGGTAATCCAAACTTATGTGG

As2.17 **F:** TCCTCGCTTCTCGACATTTT VIC 119–134 2

**R:** GCTCGAACCTTTCAAACGAA

Plop4 **F:** AAACCAAGGTCTTCTCTGCTTC PET 192-218 2

**R:** ACTCCCTCTCTTTCCATCTCT

Plop18 **F:** ATTGAAGCTGCCCTCACATT NED 178-180 1

**R**: TGTTCGGCCTCTTCTTTCTC
